# Species identification of adult ixodid ticks by Raman spectroscopy of their feces

**DOI:** 10.1186/s13071-023-06091-7

**Published:** 2024-01-30

**Authors:** Tianyi Dou, Aidan P. Holman, Samantha R. Hays, Taylor G. Donaldson, Nicolas Goff, Pete D. Teel, Dmitry Kurouski

**Affiliations:** 1 Department of Biochemistry and Biophysics, Texas A&M University, College Station, TX 77843 USA; 2 Department of Entomology, Texas A&M AgriLife Research, College Station, TX 77843 USA; 3 Department of Biomedical Engineering, Texas A&M University, College Station, TX 77843 USA

**Keywords:** Raman spectroscopy, Tick feces, Tick-infested cattle

## Abstract

**Background:**

Ticks and tick-borne diseases pose significant challenges to cattle production, thus the species identification of ticks and knowledge on their presence, abundance, and dispersal are necessary for the development of effective control measures. The standard method of inspection for the presence of ticks is the visual and physical examination of restrained animals, but the limitations of human sight and touch can allow larval, nymphal, and unfed adult ticks to remain undetected due to their small size and site of attachment. However, Raman spectroscopy, an analytical tool widely used in agriculture and other sectors, shows promise for the identification of tick species in infested cattle. Raman spectroscopy is a non-invasive and efficient method that employs the interaction between molecules and light for the identification of the molecular constituents of specimens.

**Methods:**

Raman spectroscopy was employed to analyze the structure and composition of tick feces deposited on host skin and hair during blood-feeding. Feces of 12 species from a total of five genera and one subgenus of ixodid ticks were examined. Spectral data were subjected to partial least squares discriminant analysis, a machine-learning model. We also used Raman spectroscopy and the same analytical procedures to compare and evaluate feces of the horn fly *Haematobia irritans* after it fed on cattle.

**Results:**

Five genera and one sub-genus at overall true prediction rates ranging from 92.3 to 100% were identified from the Raman spectroscopy data of the tick feces. At the species level, *Dermacentor albipictus*, *Dermacentor andersoni* and *Dermacentor variabilis* at overall true prediction rates of 100, 99.3 and 100%, respectively, were identified. There were distinct differences between horn fly and tick feces with respect to blood and guanine vibrational frequencies. The overall true prediction rate for the separation of tick and horn fly feces was 98%.

**Conclusions:**

Our findings highlight the utility of Raman spectroscopy for the reliable identification of tick species from their feces, and its potential application for the identification of ticks from infested cattle in the field.

**Graphical Abstract:**

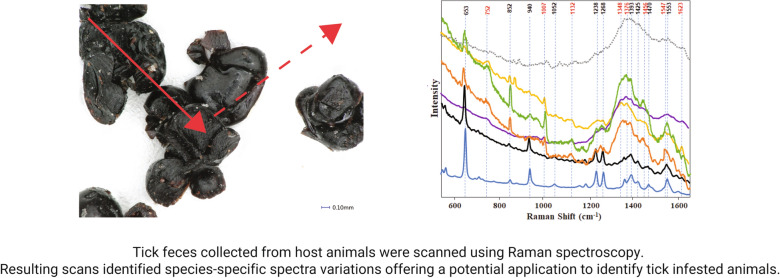

**Supplementary Information:**

The online version contains supplementary material available at 10.1186/s13071-023-06091-7.

## Background

Ticks and tick-borne diseases are major impediments to cattle production on a regional, national, and global scale [1]. Efforts to control native tick species in cattle production systems, and to prevent the introduction and spread of invasive tick species, require knowledge of their presence, abundance, and dispersal. The physical inspection of restrained animals for the presence of ticks is an important method for passive and active surveillance in regulatory programs. Surveillance data are used to identify which hosts are carrying which ticks, define boundaries of presence, and estimate rates of spread. Tick surveillance data obtained over time and space provide important information on trends in tick invasion risk associated with factors such as global trade, host diversity, climate, and land use [2].

The inspection of cattle is a key component of the Cattle Fever Tick Eradication Program of the US Animal and Plant Health Inspection Service (US Department of Agriculture), which is designed to prevent the reintroduction and re-establishment of *Rhipicephalus* (*Boophilus*) *annulatus* and *Rhipicephalus* (*Boophilus*) *microplus* from Mexico to the Southern Unites States [3]. The direct impact of these ticks on cattle production and on the transmission of *Babesia bigemina* and *Babesia bovis* were detrimental to the early development of the US cattle industry [4]. Cattle are inspected for the early detection of these ticks including those imported from Mexico, those in the Texas border counties of the permanent quarantine zone, and those sold at auction across Texas.

The distribution of the Asian long-horned tick *Haemaphysalis longicornis* has expanded to 19 US states since its initial discovery in 2017 [5]. Its role as a vector of *Theileria orientalis* Ikeda genotype in cattle [6] is of considerable concern for US cattle producers. The expected range expansion of *H. longicornis* due to the interstate movement of cattle is of concern with respect to the lack of inspection and treatment of cattle [7]. The inspection of cattle and other livestock for *H. longicornis* has been carried out within a collaborative passive surveillance network in Tennessee [8].

Biosecurity measures undertaken in cattle production systems, including inspection, treatment, and quarantine, can prevent the movement of ticks associated with the movement of cattle purchased at remote locations and/or the movement of cattle between owned/leased properties. Preventing the introduction of ticks into a production system and their spread can also prevent the spread of tick-borne pathogens.

Inspecting cattle for ticks is currently conducted by examining restrained animals by sight and touch. However, tick detection in this way is limited as there are areas of the animal’s body that cannot easily be seen or safely touched. In addition, the human sense of touch is limited to the detection of objects no smaller than approximately 8 mm [9], which limits the detection of immature ticks and some unfed adult ticks. The predilection sites for tick attachment include the head, dewlap, axillaries, belly, udder/scrotum, perineum and tailhead. The inspection process is tedious and difficult, prone to human error, and dangerous for inspectors of range cattle [10]. New methods are thus being investigated to overcome the challenges faced during the inspection of cattle for the detection of tick infestation.

Ixodid ticks blood-feed for days to weeks depending on their developmental stage (larva, nymph, or adult) and whether they drop from the host after feeding at each stage (three-host ticks) or sequentially feed through all three developmental stages (one-host ticks) before dropping from the host. While blood-feeding, ticks defecate onto the hair and skin of their host, potentially leaving evidence of their presence. We have investigated the structure and composition of feces from seven tick species using Raman spectroscopy and discovered species-specific signatures among adult ticks and evidence of signature differences between immature and adult tick feces [11].

In this study, we expanded the scope of previous work and report Raman-based spectroscopic evidence for the identification of 12 species of ixodid ticks based on the feces of their respective adults. We include an assessment of feces from the horn fly *Haematobia irritans*, an ubiquitous ectoparasite of cattle that also defecates on them while blood-feeding, and thus could confound the identification of tick species based on feces. We discuss potential applications for Raman spectroscopic findings for the identification of tick species from infested cattle and also the potential value of species-level variation in the chemical composition of tick feces.

## Methods

### Sample collection

Feces from adult ticks were collected from the host’s skin and hair or wool for cattle and sheep, respectively, using standard rearing protocols. The feces of horn flies that had fed on cattle were sampled from feces that had been deposited on glass vials. The only tick species reared on sheep were *Dermacentor variabilis* and *Ixodes scapularis*.

In all cases, protocols for the rearing of ticks and horn fly were followed as approved by the respective Institutional Animal Care and Use Committee of each contributing institution and laboratory. Samples of tick feces were collected from the feeding chambers to which individual tick species had been confined when feeding on the host animals, and included fecal matter from numerous ticks of the same species. Samples of feces were placed in clean labeled glass containers, which were kept at room temperature before they were shipped to the Tick Research Laboratory, Department of Entomology, Texas A&M AgriLife Research, College Station, Texas, where they were stored. A summary of the laboratory source and host species for the fecal samples of each tick species and the horn fly used in this study is given in Additional file 1: Table S1.

### Raman spectroscopy

A laboratory-built confocal microscope (Nikon TE-2000U) was used for confocal Raman spectroscopy. A single longitudinal mode laser diode (Necsel, CA) was utilized to generate light of a continuous wavelength (λ = 785 nm). The laser was focused on the sample by using a ×20 Nikon objective (numerical aperture 0.45). The scattered light was collected by the same objective and directed using blazed direction grating (600 groove/mm grating) at 750 nm. A long-pass filter (LP02-785RE-25; Semrock, NY) was used for filtered Rayleigh scattering before the light entered the spectrograph. The Raman-scattered (inelastically dispersed) photons were captured by a PIXIS: 400BR CCD camera (Princeton Instruments, NJ). The spectra acquisition time was between 10 and 30 s. The laser power was set to between 7 and 7.5 mW.

The tick feces samples were ground in a mortar with a pestle and placed on a glass coverslip for scanning. The horn fly feces samples were obtained from the glass vial by using a tweezer and placed on a glass coverslip for scanning. From 20 to 30 Raman spectra were obtained for each tick species, by using fresh fecal pellets of the same sample every three to five scans. In total, we scanned a total of 30 samples for reared *Amblyomma* spp. (seven *Amblyomma americanum*, 10 *Amblyomma maculatum*, seven *Amblyomma mixtum*, and six *Amblyomma tenellum*); 18 for *Dermacentor* spp. (seven *Dermacentor albipictus*, 10 *Dermacentor andersoni*, and one *Dermacentor variabilis*); four for *Haemaphysalis longicornis*; one for *Ixodes scapularis*; three for *Rhipicephalus* spp. [one *R.* (*B.*) *annulatus*, one *R.* (*B.*)* microplus*, and one *Rhipicephalus sanguineus* sensu stricto]; and two for *Haematobia irritans* (horn fly).

Raman spectroscopy is a label-free, non-invasive, and non-destructive method for the analysis of chemical composition [12]. Through the use of a focused laser, the photons interact with chemical groups of the samples, which undergo a change in energy and produce inelastically scattered photons. The vibrational frequency pattern of the molecules is affected by the energy change. Raman spectroscopy is widely used in agriculture [12, 13], forensics [14–16], and other scientific fields [17–19] for qualitative and quantitative analysis. Through the application of chemometrics, Raman spectroscopy can be used for different diagnostic models, e.g., Raman spectroscopy combined with deep learning can be used to identify different pathogenic bacteria [19]. Doty and Ledmev[20] demonstrated that Raman spectroscopy can be used to identify different animal species from the spectroscopic signatures of their blood. Farber et al. [21] and Goff et al. [22] explored the possibility of using Raman spectroscopy for the detection of Lyme disease from blood samples.

### Partial least square discriminant analysis

The classification model was based on the partial least square discriminant analysis (PLS-DA) algorithm, and conducted in MATLAB R2020a add-on PLS_Toolbox (Eigenvector Research). All the adult tick and horn fly spectra were pre-processed before being input into the model. Pre-processing included total area normalization, baseline correction (automatic Whittaker filter, asymmetry = 0.001, lambda = 100), and mean centering. Latent variables were selected depending on the performances of different models.

Chemometric algorithms including supervised and unsupervised models can be used to help analyze spectral data. Unsupervised models like principal component analysis and hierarchical cluster analysis can be used to explore the similarities and differences among data. In contrast, supervised models can be used to indicate differences, with the aim of classifying the data based on their assignment. For example, a classification model like PLS-DA, support vector machine discriminant analysis, soft independent modeling of class analogy, and other models, can be used for the qualitative analysis of the dataset [12, 23, 24].

## Results

**Fig. 1:**
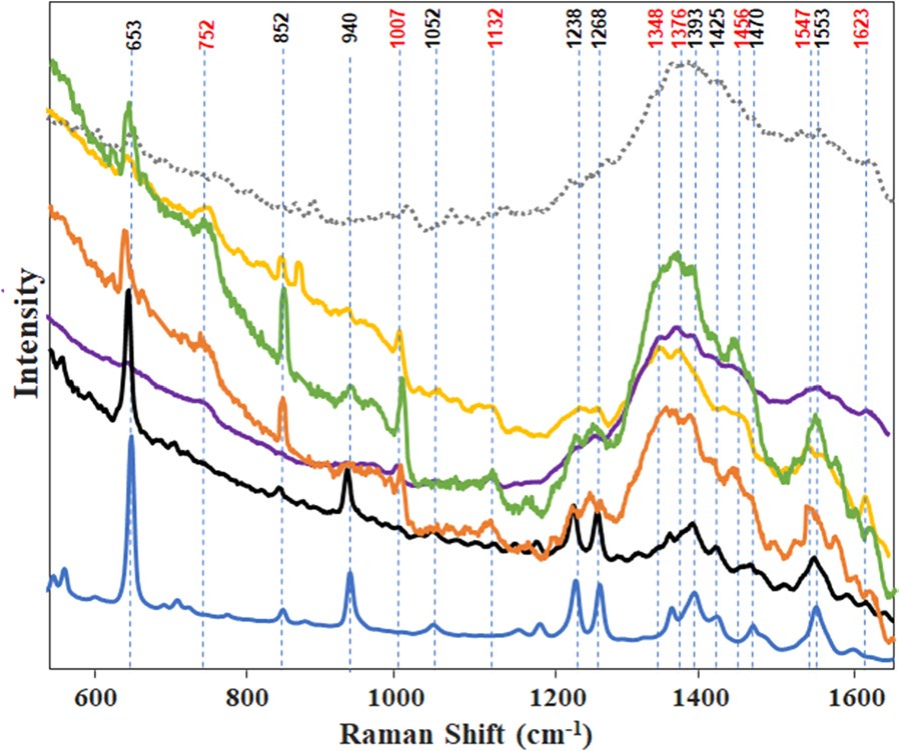
Genus- and species-level comparison of the average Raman spectra obtained for *Amblyomma* (four species; blue), *Rhipicephalus* (*Boophilus*) (two species; black)*, Rhipicephalus sanguineus* sensu stricto (orange), *Dermacentor* (three species; yellow), *Haemaphysalis longicornis* (purple), *Ixodes scapularis* (green), and *Haematobia irritans* (gray dashed line). Raman vibrational frequency bands known to be associated with blood (red) and guanine (black)

The average Raman spectra of the fecal samples of each of the 12 tick species were grouped by genus, subgenus or species as follows: *Amblyomma* (*A. americanum*, *A. maculatum*, *A. mixtum*, and *A. tenellum*); *R.* (*B.*) *annulatus* and *R.* (*B.*) *microplus*; *Dermacentor* (*D. albipictus*, *D. andersoni*, *D. variabilis*); *Haemaphysalis longicornis*; *Rhipicephalus sanguineus* sensu stricto; and *Ixodes scapularis* (Fig. 1). The overall pattern of the Raman spectra varied from near flat with low intensities (*Amblyomma*) to spectra with progressively declining intensities (*Boophilus*) to spectra with distinct peaks and troughs (*Ixodes*, *Dermacentor, Haemaphysalis,* and *Rhipicephalus*). Each spectrum had a series of peaks that aligned with the vibrational frequency bands associated with either vertebrate blood or guanine. Peaks in each Raman spectrum (Fig. 1) were identified by alignment with vibrational frequency bands, and are summarized in Table 1 to illustrate the variation in fecal content and the ratio of known blood to guanine components.

**Table 1:** Comparison of vibrational frequency bands for peaks of Raman spectra of fecal samples from 12 species of ixodid ticks

Raman spectral group	Vibrational frequency bands indicating blood (cm^−1^)	Vibrational frequency bands indicating guanine (cm^−1^)	Relative intensities of bands 653–1007
*Amblyomma* (four species)^a^	–	653, 852, 940, 1052, 1238, 1268, 1393, 1470, 1553	64.98
*Rhipicephalus* (*Boophilus*) (two species)^b^	–	653, 852, 940, 1052, 1238, 1268, 1393, 1470, 1553	4.34
*Rhipicephalus sanguineus* sensu stricto	752, 1007, 1132, 1348, 1376, 1456, 1547, 1623	653, 852, 1238, 1268, 1393, 1425, 1553	1.3
*Dermacentor* (three species)^c^	752, 1007, 1132, 1348, 1376, 1547, 1623	653, 852, 940, 1052, 1238, 1268	0.51
*Ixodes scapularis*	752, 1007, 1132, 1376, 1456	653, 852, 940, 1238, 1393, 1353	0.94
*Haemaphysalis longicornis*	752, 1007, 1348, 1376, 1547, 1623	653, 1393, 1553	0.68

^a^*Amblyomma americanum*, *Amblyomma maculatum, Amblyomma mixtum*, and *Amblyomma tenellum*

^b^*Rhipicephalus* (*Boophilus*) *annulatus* and *Rhipicephalus* (*Boophilus*) *microplus*

^c^*Dermacentor albipictus, Dermacentor andersoni*, and *Dermacentor variabilis*

We identified eight slightly variable bands that indicated the presence of components of blood and nine that indicated the presence of components of guanine in the spectra of the fecal samples of the six taxonomic groups (Table 1). All of the spectra of these six taxonomic groups had vibrational frequency bands that could be assigned to guanine, but only spectra of four of the groups (*Rhipicephalus*, *Dermacentor*, *I. scapularis,* and *H. longicornis*) were indicative of the presence of blood components. The vibrational frequency shared by all groups was that of guanine, at 653 cm^−1^. Different combinations of five of the six groups shared vibrational frequencies at 852, 1238, and 1393 cm^−1^. The vibrational frequencies at 752, 1007, and 1376 cm^−1^, which can be assigned to blood components, were shared by *Rhipicephalus sanguineus* sensu stricto,* Dermacentor*, *I. scapularis* and *H. longicornis*. To determine the ratio between blood and guanine components in each fecal sample, we used the 653 cm^−1^ vibrational frequency as a marker for guanine and 1007 cm^−1^ for components of blood. All of the spectra were baselined and normalized around 653 cm^−1^. The relative intensities at 653 and 1007 cm^−1^ indicated that feces from *Amblyomma* and the subgenus *Boophilus* were dominated by guanine, as shown by the molecular vibrations, whereas those of *Dermacentor*, *I. scapularis*, *H. longicornis* and *R. sanguineus* sensu stricto were composed of mixtures of guanine and components of blood (Table 1; Fig. 1).

**Fig. 2:**
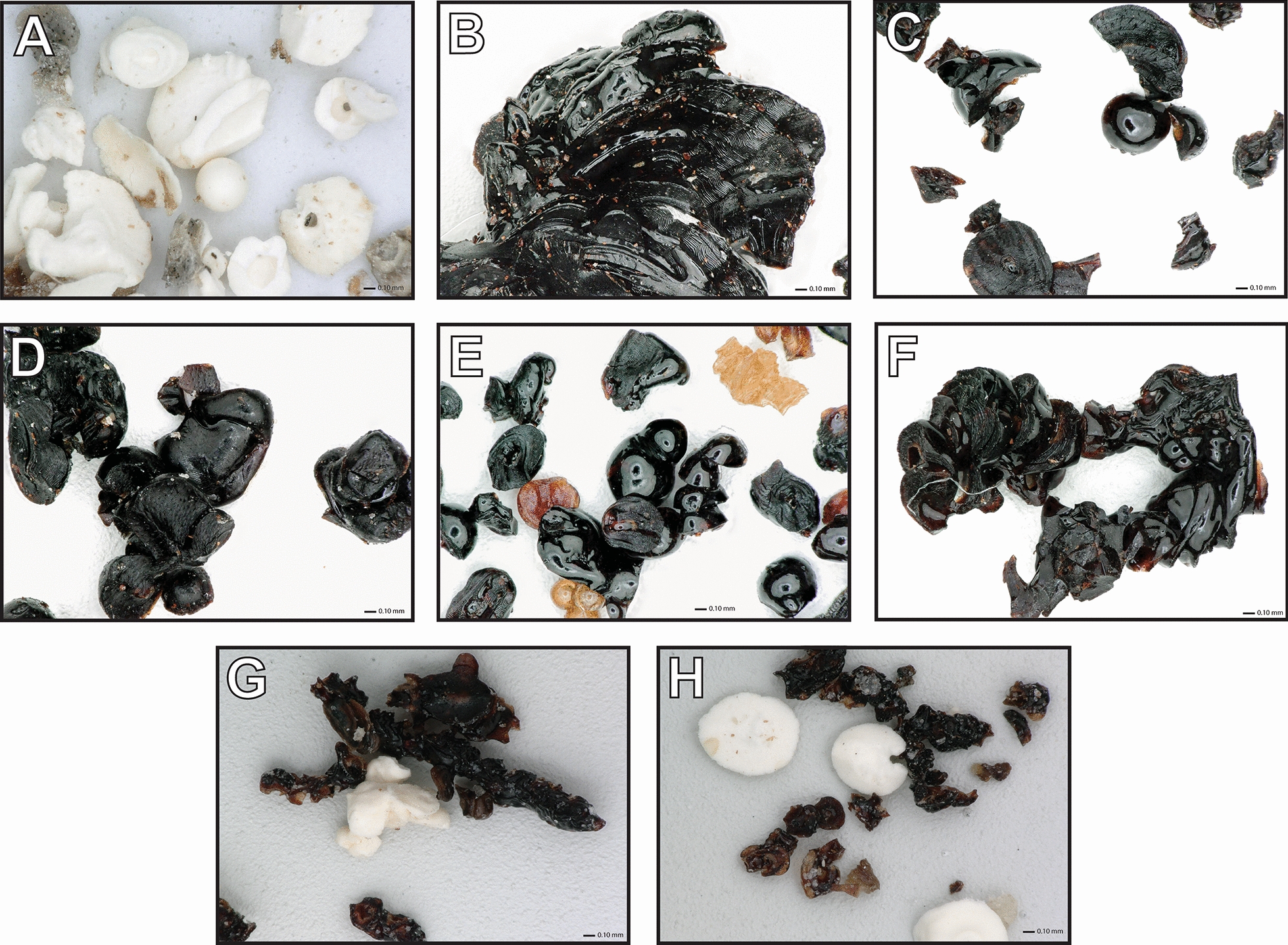
Sample variation in tick feces color for **A** *Amblyomma americanum*, **B** *Dermacentor albipictus*, **C** *Dermacentor variabilis*, **D** *Haemaphysalis longicornis*,** E** *Ixodes scapularis*, **F** *Rhipicephalus sanguineus* sensu stricto, **G** *Rhipicephalus* (*Boophilus*) *annulatus*, and **H** *Rhipicephalus* (*Boophilus*) *microplus* at ×100 magnification

There were visible differences in color bewteen tick feces, as illustrated by examples shown in Fig. 2. The feces of *D. albipictus*, *D. variabilis*, *H. longicornis*, *I. scapularis*, and *R. sanguineus* sensu stricto tended to mostly comprise shiny black particles (Table 1). These samples contained components of both blood and guanine, with a relative intensity (653–1007 cm^−1^ bands) > 0.5 but < 1.5. In comparison, the feces of *A. americanum* and the two species of the subgenus *Boophilus* contained mixtures of particles ranging from light tan to white in color. These contained guanine-like components; the relative intensity was > 4.0 (Table 1).

We next constructed a machine learning model to identify tick genera within the data set. The PLS-DA algorithm was used for the classification model (Table 2). The adult tick feces spectra were imported into the model and preprocessed. Spectra were normalized by the total area to eliminate the potential influence of tick feces color. The automatic Whittaker filter was used to baseline the spectra. Finally, the spectra underwent mean centering for the algorithm; latent variable = 13 was used in this model. The model predictions were 98.1, 92.3, 100, 99.7, 100, and 95% accurate for *Amblyomma*, subgenus *Boophilus, Rhipicephalus*, *Dermacentor*, *Haemaphysalis*, and *Ixodes*, respectively. The loading plot for the top three latent variables explained 97.2, 0.98, and 0.3% of the total predicted data (Additional file 2: Figure S1). Most of the data (97.1%) were differentiated based on their intensity even after total area normalization, since the loading plot shared a similar shape to the guanine-rich spectra. These results indicate that the genera could be identified by blood/guanine-dominated features of their feces.

**Table 2:** Accuracy of the identification of ticks to genus/subgenus level using the partial least-square discriminant analysis model for Raman spectra data of feces of 12 species of ixodid ticks

	*Amblyomma*	*Rhipicephalus* (*Boophilus*)	*Rhipicephalus*	*Dermacentor*	*Haemaphysalis*	*Ixodes*
Predicted as *Amblyomma*^a^	462	3	0	0	0	0
Predicted as *Rhipicephalus* (*Boophilus*)^b^	9	36	0	0	0	0
Predicted as *Rhipicephalus*^c^	0	0	20	1	0	1
Predicted as *Dermacentor*^d^	0	0	0	338	0	0
Predicted as *Haemaphysalis*^e^	0	0	0	0	80	0
Predicted as *Ixodes*^f^	0	0	0	0	0	19
TPR%	98.1	92.3	100	99.7	100	95

*TPR%* True positive rate (i.e., the percentage of correct predictions)

^a^*Amblyomma americanum*, *Amblyomma maculatum, Amblyomma mixtum*, and *Amblyomma tenellum*

^b^*Rhipicephalus* (*Boophilus*) *annulatus* and *Rhipicephalus* (*Boophilus*) *microplus*

^c^*Rhipicephalus* *sanguineus* sensu stricto

^d^*Dermacentor albipictus*, *Dermacentor andersoni*, and *Dermacentor variabilis*

^e^*Haemaphysalis longicornis*

^f^*Ixodes scapularis*

To further differentiate species within the genus *Dermacentor*, we conducted another analysis using the same model with the same preprocessing of data (Table 3; Additional file 3: Figure S2). The high accuracy of this model was similar to that of the previous one, and only one spectrum, that of *D. andersoni*, was misclassified as *D. albipictus*. The spectra of the three species of *Dermacentor* shared greater similarity than the other blood-rich spectra. Since the spectral data were normalized before being input into the model, the difference in spectral intensity associated with feces color would not have affected the model prediction.

**Table 3:** Accuracy of all predicted ixodid tick taxa versus horn fly *Haematobia irritans*, and accuracy of the identification of three species within the genus *Dermacentor*, based on identification of the source of the feces using partial least-square discriminant analysis models for Raman spectra data

	Tick	Fly
Predicted as tick feces	954	0
Predicted as fly feces	15	40
TPR%	98.45	100

	*Dermacentor albipictus*	*Dermacentor andersoni*	*Dermacentor variabilis*
Predicted as *D. albipictus*	180	1	0
Predicted as *D. andersoni*	0	183	0
Predicted as *D. variabilis*	0	0	20
TPR%	100%	99.28	100

**Fig. 3:**
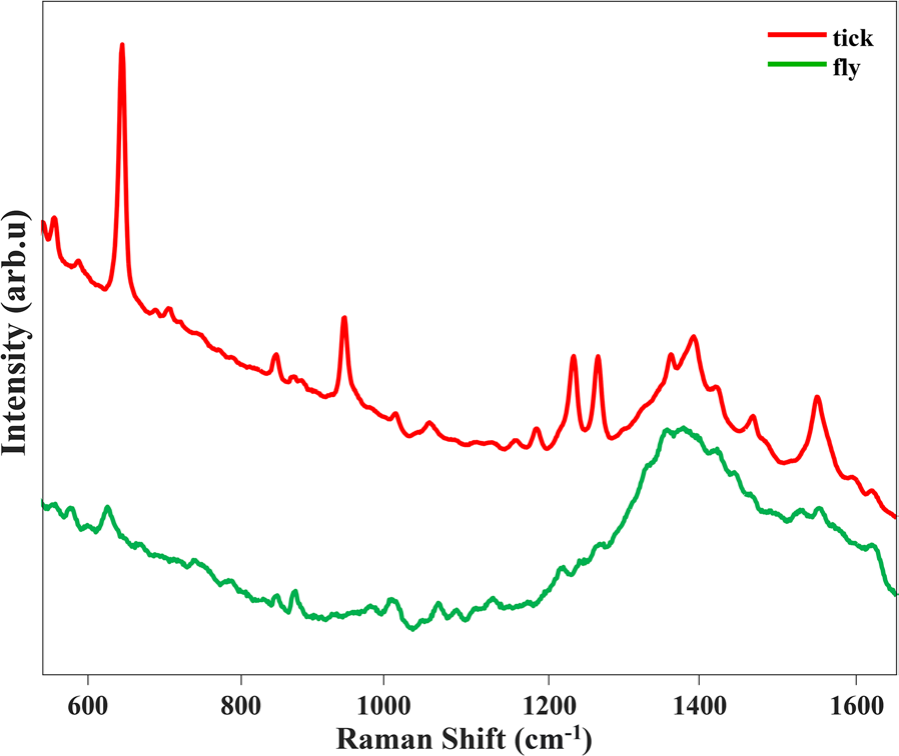
Averaged Raman spectra of fecal samples from 12 species of ixodid ticks (red) compared to those of *Haematobia irritans*

The Raman spectra of the horn fly feces exhibited higher similarity to the spectra of feces of *Dermacentor* and *Ixodes*, which were characterized by high levels of blood components (Table 1). The broad peak at around 1350–1400 cm^−1^ includes the vibrational frequencies at 1348 and 1376 cm^−1^ that originate from blood and the vibrational frequency that can be assigned to guanine, at 1393 cm^−1^ (Fig. 3). Next, we assigned our data to one of two classes (tick or fly) and input them into our PLS-DA classification model for prediction. The preprocessing method for these spectral data was the same as that used for the previous model. Our model had an overall 98% true prediction rate for the separation of tick from fly feces (Table 3). All 40 fly feces spectra were correctly predicted, but 15 of the 969 spectra for tick feces were incorrectly classified as fly feces spectra. This small proportion of misclassified data may have resulted from the presence of vibrational frequency bands of blood components in the spectra. Our loading plot (Additional file 4: Figure S3) was 98.4% for the total data. However, most of the data were differentiated based on features of the guanine vibrational frequency band. In addition to the main features of this, a peak at 1007 cm^−1^ that was associated with a blood-like feature in the top three latent variables, indicated that the ratio between peaks was also important with respect to the differentiation of samples by the model.

## Discussion

The application of Raman spectroscopy to measure the constituents of feces from adult ixodid ticks that have engorged on cattle or sheep blood provides a basis for the evidence-based species-specific identification of ticks that have infested animals. Tick feces deposited on animals during blood-feeding may provide evidence of tick infestation. The Raman spectra of feces from 12 tick species revealed unique vibrational frequency bands that indicated the presence of components of blood, guanine, and as yet unidentified constituents of the feces. Vibrational frequency bands associated with blood or blood-like components [11, 21, 25, 26] and guanine or guanine-like components [11, 27, 28] have been previously reported.

The PLS-DA models enabled the prediction of ticks to genus level, with an average accuracy of identification of 97% when differentiating between the six taxonomic groups in the present study, and over 99% accuracy when differentiating between three species of the genus *Dermacentor*. We previously reported [11] a true positive rate for species-level classification of four *Amblyomma* species, *R. (B.) annulatus* and *R. (B.) microplus* as 100%. Ten of the 12 tick species in the present study are representative of ticks commonly found on cattle in Texas, and the identification of cattle infestations with *R.* (*B.*) *annulatus* or *R.* (*B.*) *microplus* is essential for the state-federal Cattle Fever Tick Eradication Program for the prevention of bovine babesiosis [4]. The lowest percentage of correct predictions at the genus/subgenus level in this study was 92.3% for *Rhipicephalus (Boophilus*) due to the three spectra that were erroneously predicted to indicate *Amblyomma*. As South Texas cattle are known to be co-infested with multiple ixodid species, species identification by Raman spectroscopy of tick feces has to be both highly specific and sensitive. The discovery that *Haemaphysalis longicornis* and *Dermacentor andersoni* can be clearly distinguished from all 10 of the other species by using Raman spectroscopy of fecal samples is similarly important due to their respective geographic ranges in the US and their roles as vectors of the pathogens that cause bovine theileriosis [6] and bovine anaplasmosis [29], respectively.

The PLS-DA model predictions differentiating *H. irritans* feces from those of all 12 species of ixodid ticks was > 95%, indicating that the potential for confounding the identification of tick feces with those of the horn fly may be minimal. This is of significance because of the blood-feeding behavior of this fly and its overlapping distribution with those of the 12 species of ticks examined here. The spectra of *H. irritans* feces were not similar to those of the blood components of the ticks (e.g., *Dermacentor* spp.). A possible explanation for this is that the amount of blood imbibed per feeding event is 1.71 mg for *H. irritans* [30], while, for example, an adult female *D. andersoni* ingests up to 4000 mg per feeding event [31], hence the likelihood of more intense spectral signals from the latter’s feces after blood-feeding [30, 31]. The digestive processes of ticks and horn flies also differ [32, 33], which may also explain why there are differences in the spectral signals of their feces. Other blood-feeding arthropods that need to be investigated with respect to potentially confounding identification include the cattle-sucking lice *Linognathus vituli*, *Haematopinus eurysternus*, and *Solenopotes capillatus*. Two other arthropods, the spinose ear tick *Otobius megnini* and the stable fly *Stomoxys calcitrans*, will defecate on cattle during blood-feeding. However, they limit their attachment and blood-feeding to the inner portion of the outer ear, and lower portion of the legs, respectively, where other ticks do not normally attach.

The primary constituents of nitrogenous waste in ticks are generally recognized to be guanine-rich products, whereas the primary nitrogenous waste product in insects is uric acid [34]. Guanine-rich feces play a role in water conservation, which is important for the water homeostasis of ticks. Hamdy [35] analyzed the feces of eight species of *Haemaphysalis*, and of *D. andersoni*, that had fed on rabbit, sheep or calves by using solvent extraction, spectrophotometric, and electrophoretic methods [36]. All of the samples were from mated females, with the exception of one unmated female and one nymph of *D. andersoni*. The percentage content in the feces across all samples was 1.4–9.3% for guanine, 2.0–15% for another purine, 0.1–1.4% for hematin, and 5.0–68.0% for protein. The protein content varied with respect to host blood meal, i.e., it was 13 times higher for feces of *D. andersoni* that fed on sheep than for the same species of tick when it fed on rabbit. Furthermore, there was an inverse relationship between percentage protein and percentage hematin across the nine species evaluated, and for the one to nine amino acids that were found to be present according to species and blood meal source. The production and composition of tick feces may vary with tick species-host combinations. However, little additional knowledge has been gained on the physiology and biochemistry of tick Malpighian tubules and their rectal sac, and thus the excretory processes and fecal products of ticks, since the beginning of the twenty-first century [32].

The findings reported herein support the potential application of Raman spectroscopy for the identification of ixodid ticks from their feces, and for the evidence-based study of tick species of infested cattle, and to increase our knowledge of excretory processes in ticks. In the future, we will undertake the complete chemical analyses of the Raman spectra of tick feces using liquid and gas chromatography with mass spectroscopy to increase component identification, analyze the composition of feces, and to undertake quantitative comparisons within and across all 12 of these tick species. We will also evaluate potential within-species variation of the Raman spectra of the feces of ticks with respect to their geographic distribution, genetic characteristics of their strains, developmental stages (larva, nymph to adult) between three-host and one-host species, and differences between blood meals of different hosts. Future work should also include the identification of tick species from mixed-species fecal samples, the variable dynamics of tick feces production, and their deposition on cattle, and the evaluation of sampling techniques and sampling practices.

## Conclusions

The identification of ticks that infest cattle by using scratch inspection is an essential part of tick surveillance, but is acknowledged to be an imperfect method. New methods that could be reasonably applied to improve inspections could potentially improve the surveillance of tick infestation in animals. Raman spectroscopy of ixodid tick feces deposited on host animals during blood-feeding may offer an additional method of tick surveillance. The spectra of the feces of the 12 species of ixodid ticks examined here showed species-specific differences in the relative intensities and composition of known vibrational frequency bands for blood-like and guanine-like constituents, and model algorithms based on partial least-square discriminant analysis showed a high level of accuracy for the prediction of rates. The results presented here should encourage further research to improve the identification of the components of tick feces and methods for their sampling, and to increase our knowledge on the causes of within-species variation that may affect spectral data and data interpretation.

### Supplementary Information

**Additional file 1: Table S1.** Summary of colony sources for feces from 12 species of ixodid ticks and the horn fly.

**Additional file 2: Figure S1.** Loading plot of the top three latent variables (LVs) for the tick genera model that includes Raman spectra from feces of four species of *Amblyomma*; three species of *Dermacentor*; *Haemaphysalis longicornis;* *Ixodes scapularis*; and three species of *Rhipicephalus*.

**Additional file 3: Figure S2.** Averaged Raman spectra for *Dermacentor albipictus* (green), *Dermacentor andersoni* (orange), and *Dermacentor variabilis* (blue).

**Additional file 4: Figure S3.** Averaged Raman spectra for *Rhipicephalus *(*Boophilus*)* annulatus* (blue), *Rhipicephalus *(*Boophilus*)* microplus *(green), and *Rhipicephalus sanguineus* sensu stricto (orange).

## Data Availability

The spectral data that support the findings of this study are not publicly available due to their potential commercial use, but may be made available by the corresponding author upon reasonable request. The data are under controlled access storage at Texas A&M AgriLife Research.

## Abbreviations

PLS-DA: Partial least squares discriminant analysis
US: United States
