# Supplementary material for: Species identification of adult ixodid ticks by Raman spectroscopy of their feces
Source: Parasit Vectors. 2024 Jan 30;17:43. doi: 10.1186/s13071-023-06091-7 (PMC10825978; doi:10.1186/s13071-023-06091-7)
Supplement: Supplementary file 1 — Additional file 1: Table S1. Summary of colony sources for feces from 12 species of ixodid ticks and the horn fly. [file 13071_2023_6091_MOESM1_ESM.docx]

Table S1. Summary of colony sources for feces from 12 species of ixodid ticks and the horn fly.

| **Arthropod Species** | **Host Species** | **Feces Sample Source** |
| --- | --- | --- |
| *Amblyomma americanum* | *Bos taurus* | Tick Research Laboratory, Department of Entomology, Texas A&M AgriLife Research, College Station, TX |
| *Amblyomma maculatum* | *Bos taurus* | Tick Research Laboratory, Department of Entomology, Texas A&M AgriLife Research, College Station, TX |
| *Amblyomma mixtum* | *Bos taurus* | Tick Research Laboratory, Department of Entomology, Texas A&M AgriLife Research, College Station, TX |
| *Amblyomma tenellum* | *Bos taurus* | Tick Research Laboratory, Department of Entomology, Texas A&M AgriLife Research, College Station, TX |
| *Dermacentor albipictus* | *Bos taurus* | Tick Research Laboratory, Department of Entomology, Texas A&M AgriLife Research, College Station, TX |
| *Dermacentor andersoni* | *Bos taurus* | USDA-ARS Animal Disease Research Unit 3003 ADBF, WSU, Pullman, WA |
| *Dermacentor variabilis* | *Ovis aries* | Tick Rearing Facility, Department of Entomology & Plant Pathology, Oklahoma State University, Stillwater, OK |
| *Haemaphysalis longicornis* | Bos taurus | USDA-ARS Animal Disease Research Unit 3003 ADBF, WSU, Pullman, WA |
| *Ixodes scapularis* | *Ovis aries* | Tick Rearing Facility, Department of Entomology & Plant Pathology, Oklahoma State University, Stillwater, OK |
| *Rhipicephalus (Boophilus) annulatus* | *Bos taurus* | USDA-ARS Cattle Fever Tick Research Laboratory, Edinberg, TX |
| *Rhipicephalus (Boophilus) microplus* | *Bos taurus* | USDA-ARS Cattle Fever Tick Research Laboratory, Edinberg, TX |
| *Rhipicephalus sanguineus* sensu stricto | *Ovis aries* | Tick Rearing Facility, Department of Entomology & Plant Pathology, Oklahoma State University, Stillwater, OK |
| *Haematobia irritans* | *Bos taurus* | USDA-ARS Knipling-Bushland U.S. Livestock Insects Research Laboratory, Kerrville, TX  Pia Olafson |
