# Supplementary figures and images for: Species identification of adult ixodid ticks by Raman spectroscopy of their feces
Source: Parasit Vectors. 2024 Jan 30;17:43. doi: 10.1186/s13071-023-06091-7 (PMC10825978; doi:10.1186/s13071-023-06091-7)

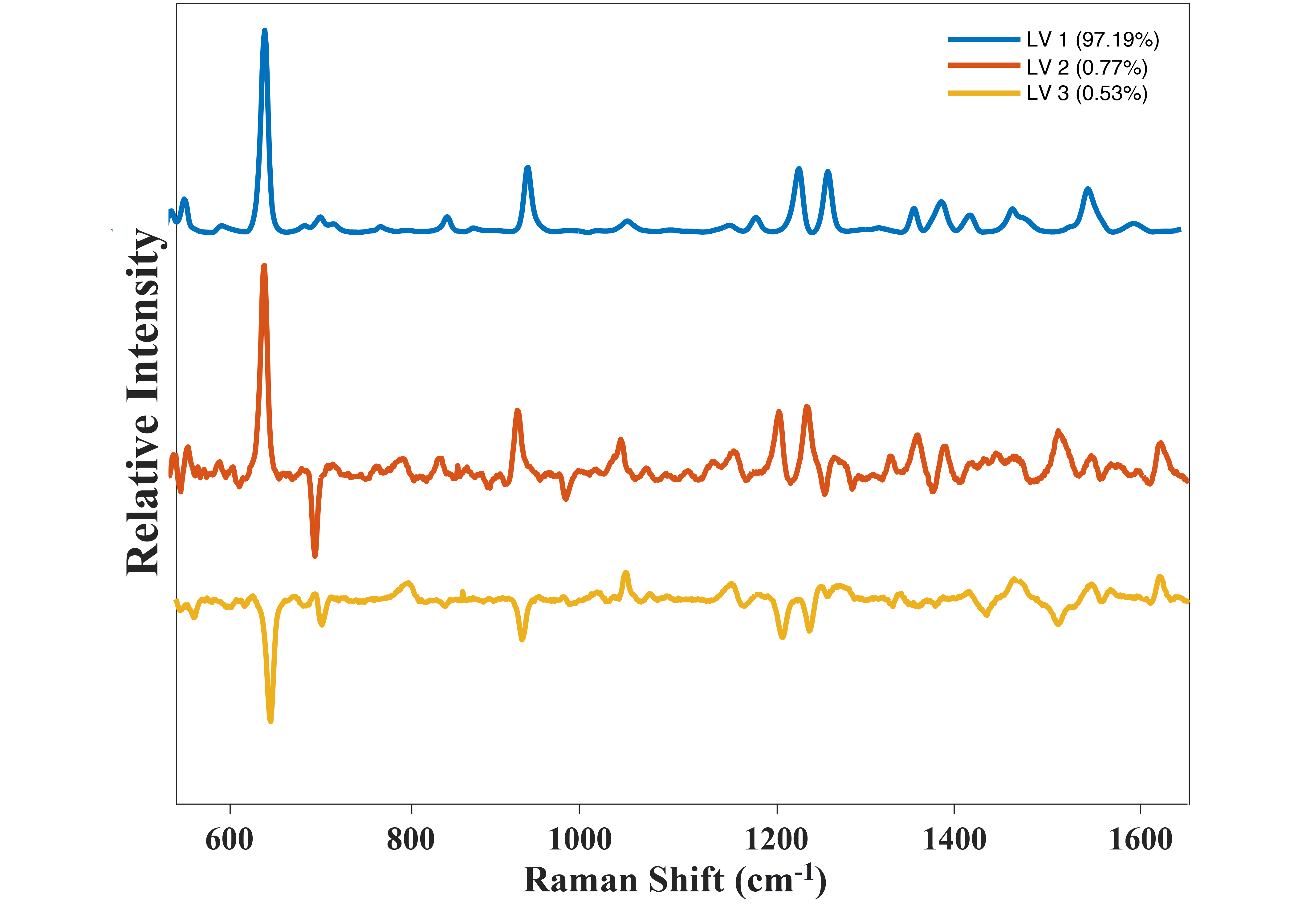

Supplement: Supplementary file 2 — Additional file 2: Figure S1. Loading plot of the top three latent variables (LVs) for the tick genera model that includes Raman spectra from feces of four species of Amblyomma; three species of Dermacentor; Haemaphysalis longicornis; Ixodes scapularis; and three species of Rhipicephalus. [file 13071_2023_6091_MOESM2_ESM.tiff]

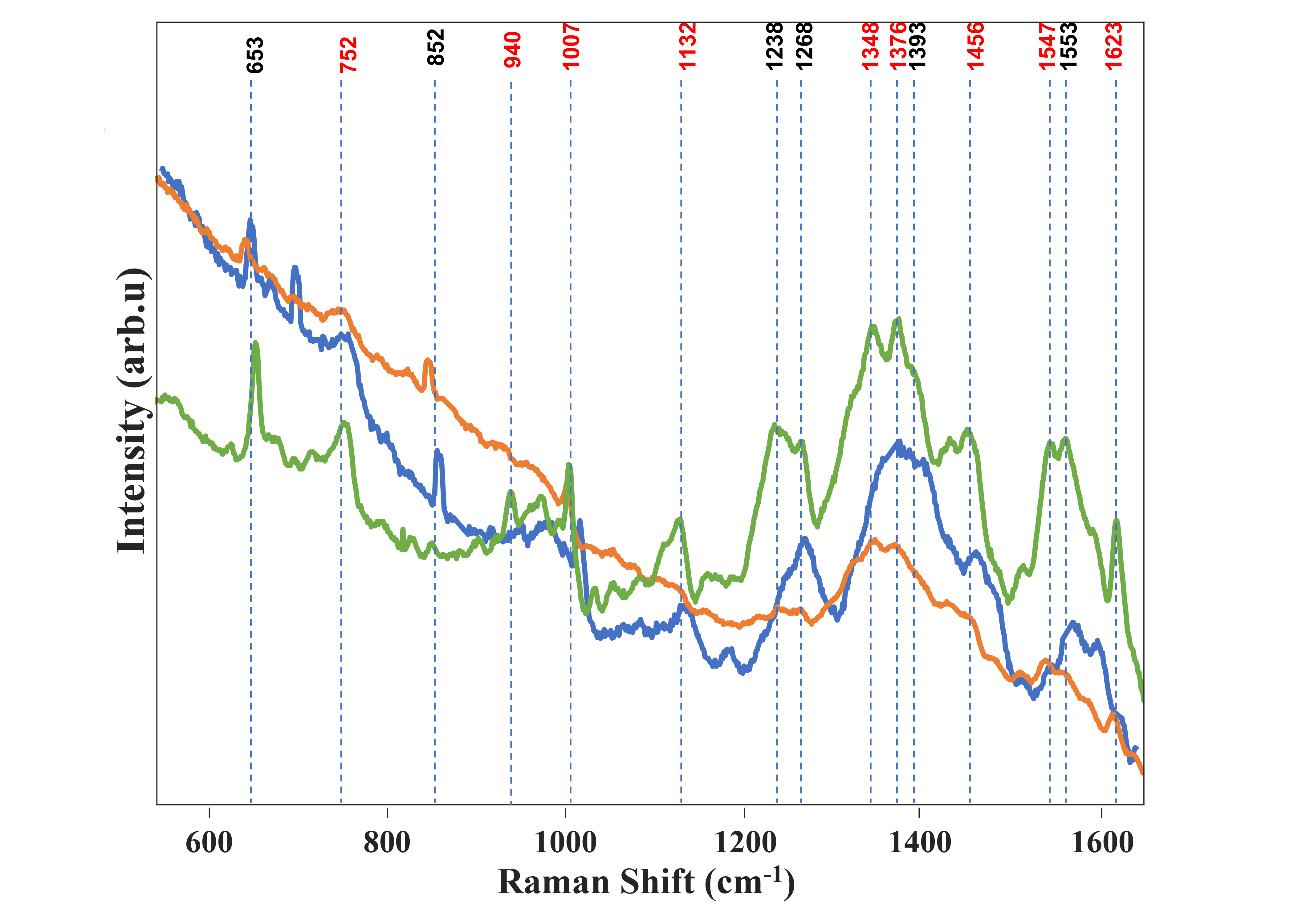

Supplement: Supplementary file 3 — Additional file 3: Figure S2. Averaged Raman spectra for Dermacentor albipictus (green), Dermacentor andersoni (orange), and Dermacentor variabilis (blue). [file 13071_2023_6091_MOESM3_ESM.tiff]

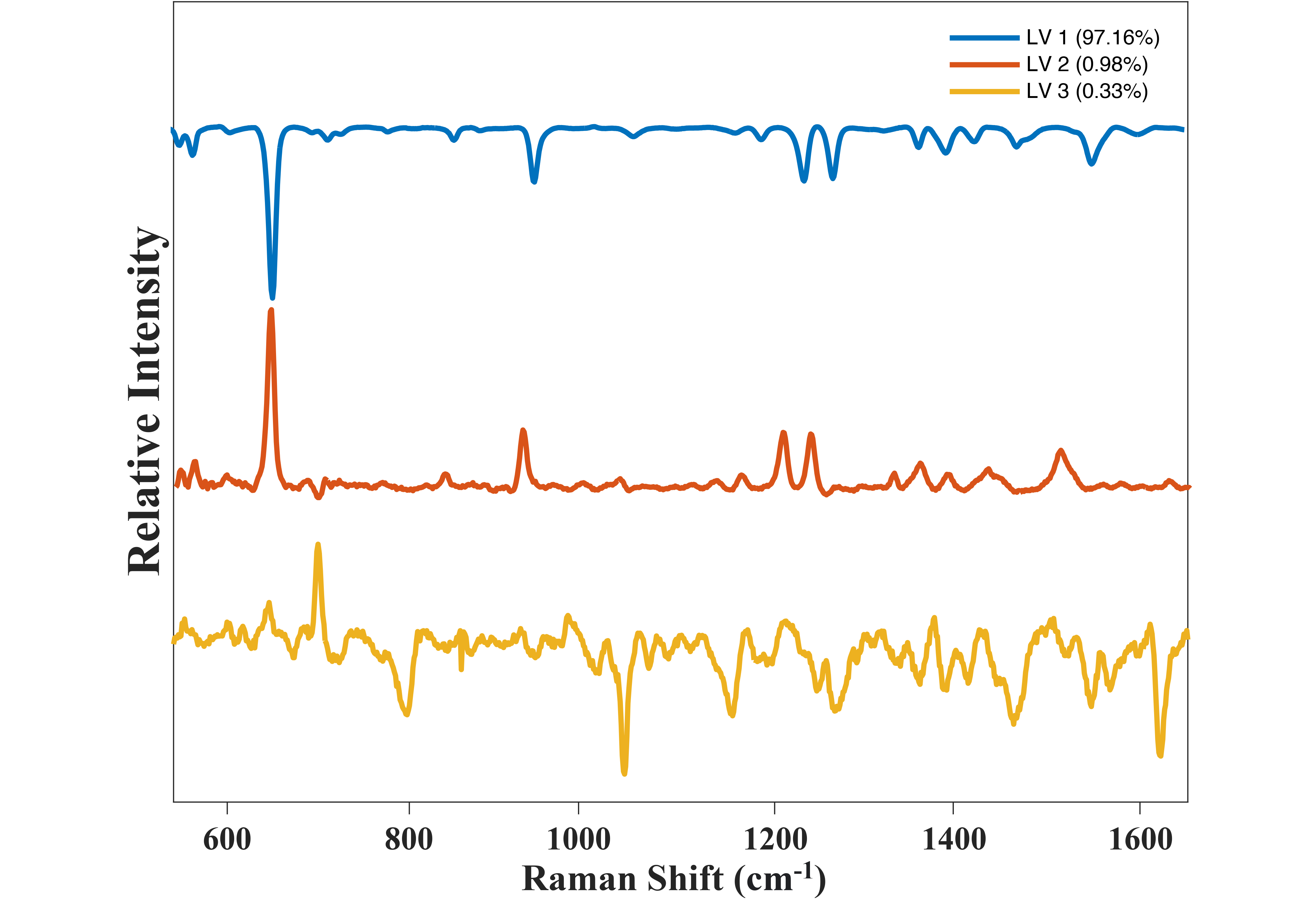

Supplement: Supplementary file 4 — Additional file 4: Figure S3. Averaged Raman spectra for Rhipicephalus (Boophilus) annulatus (blue), Rhipicephalus (Boophilus) microplus (green), and Rhipicephalus sanguineus sensu stricto (orange). [file 13071_2023_6091_MOESM4_ESM.tiff]
